# Molecular and phenotypic characterization of *Streptococcus uberis* isolates from bovine mastitis milk in South Korea

**DOI:** 10.1128/spectrum.00772-26

**Published:** 2026-06-22

**Authors:** Hye Jeong Kang, Ju-Yeon You, Seung Hoe Kim, Yun Sang Cho, Hyun-Mi Kang

**Affiliations:** 1 Bacterial Disease Division, Animal and Plant Quarantine Agency 65359, Gimcheon-si, Republic of Korea; Michigan State University, East Lansing, Michigan, USA

**Keywords:** antimicrobial resistance, bovine mastitis, *Streptococcus uberis*, multilocus sequence typing, virulence factors, biofilm

## Abstract

*Streptococcus uberis*, a major environmental pathogen, is frequently linked to recurrent or persistent intramammary infections, posing a challenge to mastitis control in dairy cattle. In this study, we characterized 112 *S. uberis* isolates recovered from bovine mastitis milk samples collected across South Korea between 2019 and 2024. Multilocus sequence typing revealed substantial genetic diversity, with all sequence types being newly assigned, and no evidence of a dominant clonal lineage. High levels of resistance to tetracycline (81.3%), erythromycin (75.9%), and pirlimycin (75.0%) were observed. Notably, 74.1% of the isolates were classified as multidrug-resistant. The corresponding resistance genes *tetO* (82.1%) and *ermB* (79.5%) were commonly detected and showed strong concordance with resistance phenotypes. Virulence-associated genes, including *sua*, *mtuA*, *oppF*, *skc*, and *pauA*, were widely detected (89.3–100%). All isolates were capable of biofilm formation, although the majority demonstrated weak biofilm-formation ability. This study provides important insights into the epidemiology and pathogenic potential of bovine mastitis-associated *S. uberis* isolates in South Korea and underscores the need for continued surveillance and prudent antimicrobial use to formulate effective mastitis-control strategies.

**IMPORTANCE:**

*Streptococcus uberis* is a leading environmental cause of bovine mastitis worldwide, yet region-specific molecular epidemiological data remain limited in South Korea. To our knowledge, this study represents the first nationwide molecular characterization of mastitis-associated *S. uberis* isolates in South Korea, integrating MLST-based population analysis, antimicrobial susceptibility testing, resistance gene detection, and virulence profiling. The identification of exclusively novel sequence types indicates substantial genetic diversity within the regional population. The high prevalence of tetracycline and MLS-type resistance, closely associated with *tetO* and *ermB*, highlights the importance of continued monitoring of antimicrobial resistance in the dairy production. Additionally, the widespread detection of core virulence-associated genes and universal biofilm-forming capacity indicates conserved characteristics among mastitis-associated isolates. These findings provide baseline data to support future surveillance and control strategies targeting bovine mastitis-associated *S. uberis*.

## INTRODUCTION

Bovine mastitis is a major infectious disease affecting dairy cattle worldwide (1–3). It results in substantial economic losses owing to decreased milk production, impaired milk quality, and increased costs associated with treatment and culling (1–3). The implementation of mastitis-control programs has markedly reduced the incidence of mastitis caused by classical contagious pathogens (3, 4). However, these programs have been less effective against infections caused by environmental pathogens (3, 4). Consequently, environmental pathogens, including *Streptococcus uberis*, have emerged as leading causes of bovine mastitis (4).

*Streptococcus uberis* is a gram-positive, catalase-negative, environmental streptococcal species and a well-recognized etiological agent of bovine mastitis (5). It is commonly found in bedding materials, soil, water, and on the skin surface of cattle. Intramammary infection is thought to occur primarily through environmental exposure, although limited cow-to-cow transmission has also been reported (2, 3, 5). Moreover, *S. uberis* is frequently associated with recurrent or persistent intramammary infections, further highlighting its importance in mastitis epidemiology (2).

Molecular epidemiological approaches have been widely used to assess strain dissemination and to inform infection-control strategies (6, 7). Several genotyping methods, including multilocus sequence typing (MLST), pulsed-field gel electrophoresis (PFGE), and whole-genome sequencing (WGS), have been applied to investigate population structure and genetic diversity in *S. uberis* (6–9). Among these methods, MLST is widely used for characterizing the population structure and genetic relatedness of *S. uberis* isolates (9–11). Previous MLST-based studies have shown that *S. uberis* populations are often characterized by marked genetic diversity, with numerous distinct sequence types (STs) and generally limited evidence for dominant clone dissemination across regions (11–15). Moreover, the population structure of *S. uberis* has been shown to vary substantially by geographic region, highlighting the need for region-specific molecular epidemiological data (11).

The ability of *S. uberis* to cause intramammary infection is influenced by a range of virulence-associated traits that facilitate colonization and persistence within the bovine mammary gland (8, 16). Previous studies in *S. uberis* have identified numerous virulence-associated genes that are involved in adhesion, nutrient acquisition, immune evasion, and host tissue interaction (16–19). These genes reflect the capacity of *S. uberis* to adapt to and survive in the mammary environment (16–19). In addition to these genetic factors, biofilm formation has been recognized as an important phenotypic characteristic of *S. uberis* (2, 8). Biofilm formation can promote bacterial attachment and persistence within the mammary environment (2). It has been associated with recurrent or persistent intramammary infections, particularly in the context of environmental exposure and host defense pressures (17). However, studies integrating virulence-associated gene profiles with phenotypic traits such as biofilm formation remain limited, particularly for mastitis-associated *S. uberis*.

In South Korea, *S. uberis* has been reported as a major causative agent of bovine mastitis (20). Despite the recognized importance of *S. uberis* in bovine mastitis, studies from South Korea have largely focused on antimicrobial resistance, leaving population structure, virulence-associated gene profiles, and biofilm-formation ability relatively underexplored. Therefore, the objective of the present study was to characterize *S. uberis* isolates recovered from bovine mastitis milk samples collected across South Korea. For this purpose, we integrated MLST-based population analysis with antimicrobial susceptibility testing, resistance and virulence gene detection, and biofilm-formation ability assessment. This integrated approach aims to provide additional insights into the epidemiology and pathogenic potential of *S. uberis* associated with bovine mastitis in South Korea.

## RESULTS

### STs of *S. uberis*

**Fig 1:**
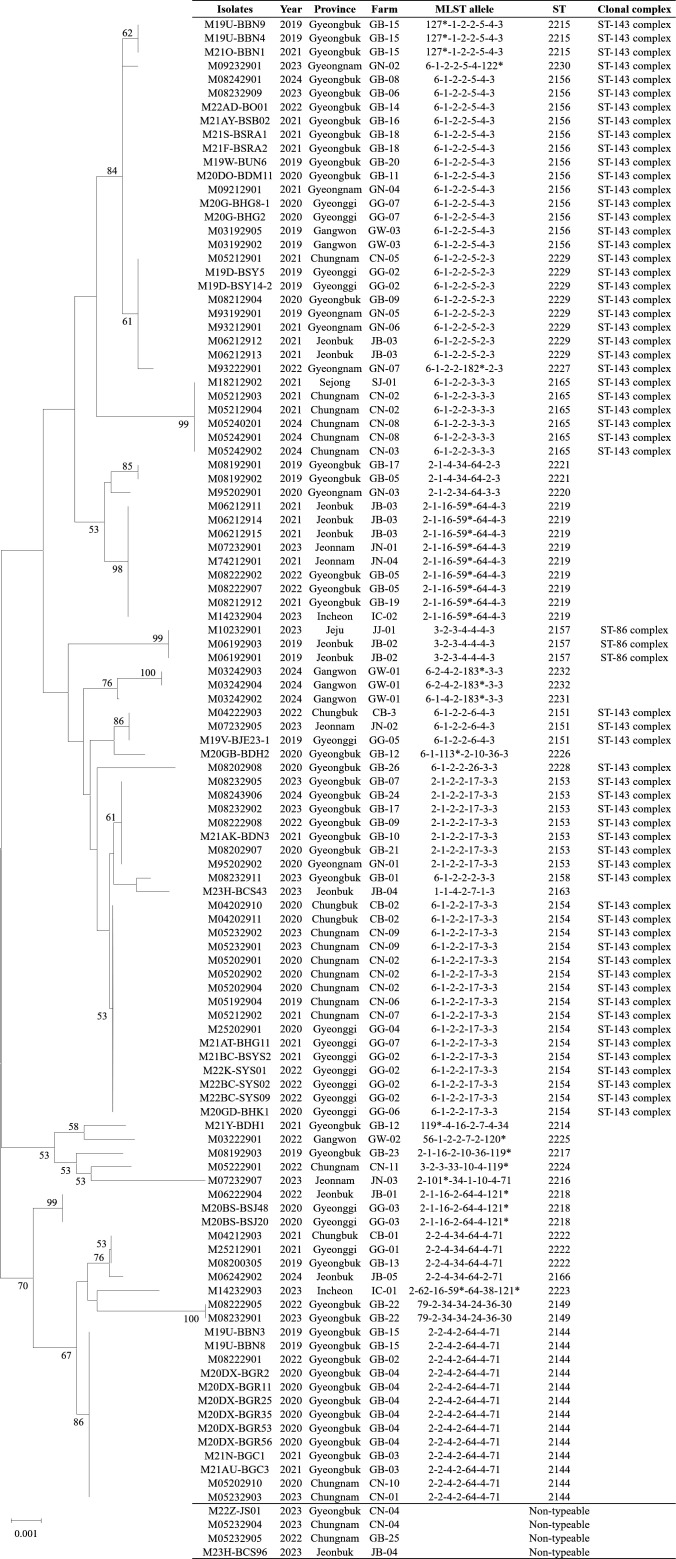
MLST-based phylogenetic tree of *Streptococcus uberis* isolates from bovine mastitis milk in South Korea. The tree was constructed using the neighbor-joining method based on the Tamura–Nei model in MEGA11 with 1,000 bootstrap replicates. Information on the isolate identifier, province, year of isolation, MLST allele profile, sequence type (ST), and clonal complex (CC) is included in the tree. The scale bar represents the number of substitutions per site. * indicates that these MLST alleles were newly identified in this study. MLST, multilocus sequence typing.

A total of 112 *S. uberis* isolates collected from various regions in South Korea were included in this study. MLST was performed to characterize the genetic diversity of isolates (Fig. 1). Four isolates were non-typeable, and the remaining 108 typeable isolates belonged to sequence types that were not previously registered in the PubMLST database. Several new allele types were identified, including *arcC* (119, 127), *ddl* (101), *gki* (113), *recP* (59), *tdk* (182, 183), and *yqiL* (119, 120, 121, 122). These alleles were submitted to PubMLST. A total of 30 novel STs were registered (ST2144, ST2149, ST2151, ST2153, ST2154, ST2156, ST2157, ST2158, ST2163, ST2165, ST2166, and ST2214–ST2232). Among these, ST2154 (16 isolates, 14.3%), ST2144 (13 isolates, 11.6%), and ST2156 (13 isolates, 11.6%) were the most common. These were followed by ST2219 (nine isolates, 8.0%), ST2229 (eight isolates, 7.1%), ST2153 (seven isolates, 6.3%), and ST2165 (six isolates, 5.4%). ST2151, ST2157, ST2215, ST2218, and ST2222 were detected in three isolates each (2.7%), whereas ST2149, ST2221, and ST2232 were found in two isolates each (1.8%). The remaining STs (ST2158, ST2163, ST2166, ST2214, ST2216, ST2217, ST2220, ST2223, ST2224, ST2225, ST2226, ST2227, ST2228, ST2230, and ST2231) were identified in one isolate each (0.9%). Based on the global clonal complex (GCC) analysis, 60 isolates (55.6%) belonged to the ST-143 complex and 3 (2.8%) to the ST-86 complex, while the remaining 45 isolates (41.7%) did not cluster into any defined GCC.

**Fig 2:**
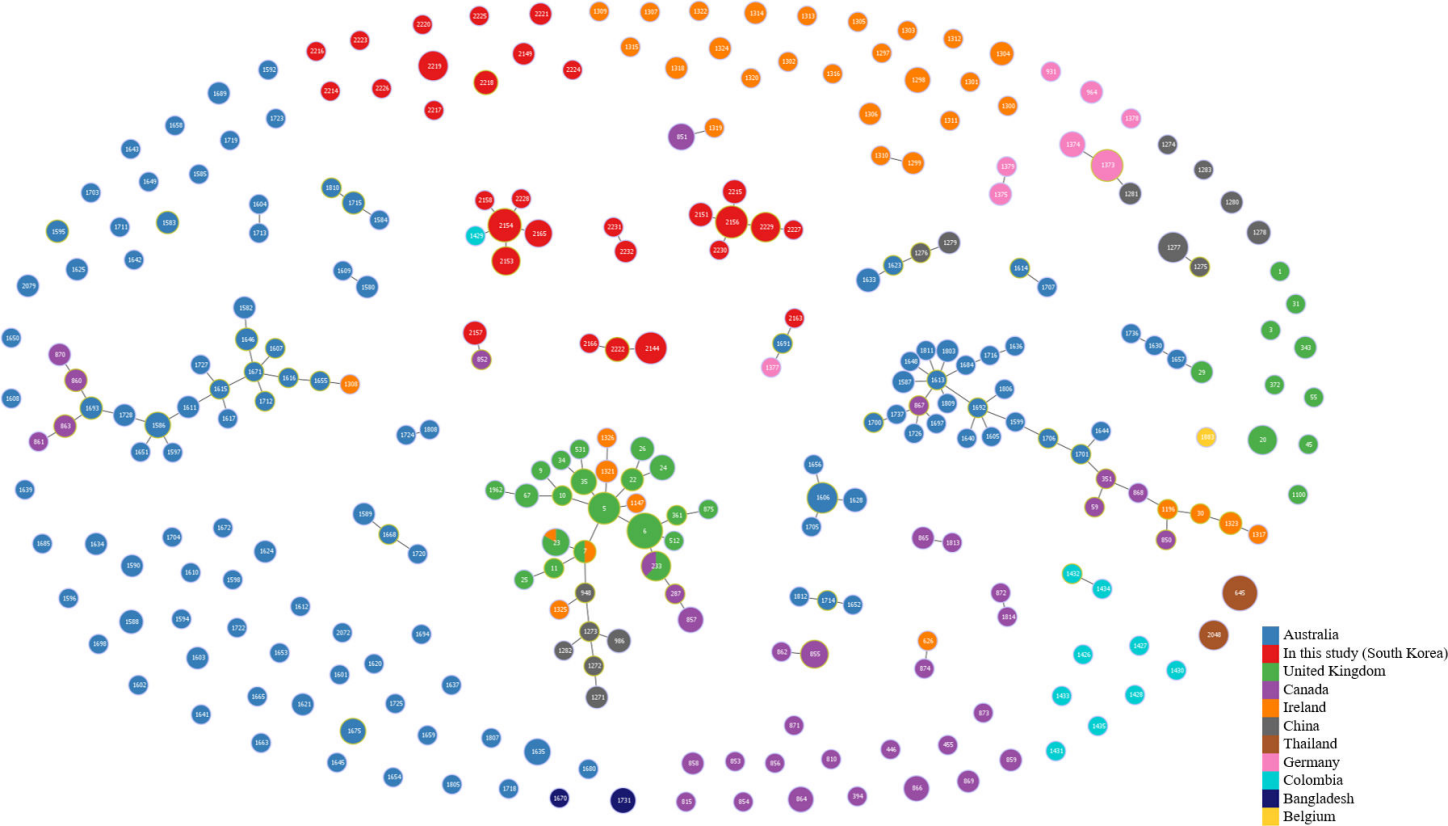
Minimum spanning tree of *Streptococcus uberis* isolates from bovine mastitis milk based on MLST allelic profiles. The data set comprised data on 108 isolates obtained from bovine mastitis milk in this study and data on 467 publicly available isolates downloaded from the PubMLST database. Each node represents a distinct sequence type (ST), and the node size is proportional to the number of isolates assigned to that ST. Edges denote single-locus variant relationships. The node colors indicate the country of isolation. MLST, multilocus sequence typing.

In the single-locus variant (SLV)-based global goeBURST minimum spanning tree, the STs from this study appeared as several small clusters or as singletons (Fig. 2). Most STs from this study did not show SLV links to previously reported global STs.

### Antimicrobial susceptibility of *S. uberis*

To determine the antimicrobial resistance patterns of the *S. uberis* isolates, minimum inhibitory concentration (MIC) testing was performed (Table 1). Twenty (17.9%) isolates were susceptible to all tested antimicrobials. The highest resistance rate was observed for tetracycline (81.3%, *n* = 91), followed by erythromycin (75.9%, *n* = 85) and pirlimycin (75.0%, *n* = 84). Only four isolates (3.6%) were resistant to oxacillin + 2% NaCl. All isolates were susceptible to ampicillin, ceftiofur, cephalothin, penicillin, and penicillin/novobiocin. The most common resistance pattern was erythromycin/pirlimycin/tetracycline, identified in 71.4% (*n* = 80) of the isolates. Multidrug resistance was observed in 74.1% (*n* = 83) of the isolates.

**TABLE 1:** Minimum inhibitory concentrations (MICs) and resistance rates of *Streptococcus uberis* isolated from bovine mastitis milk^*a*^

Antimicrobial classes	Antimicrobials	MIC_50_ (µg/mL)	MIC_90_ (µg/mL)	No. of resistant isolates (%)
β-Lactams	Ampicillin	0.25	0.5	0 (0)^c^
	Ceftiofur	1	2	0 (0)^c^
	Cephalothin	≤2	≤2	0 (0)^c^
	Oxacillin + 2% NaCl	≤2	≤2	4 (3.6)^b^
	Penicillin	0.25	0.5	0 (0)^c^
β-Lactam and aminocoumarin combination	Penicillin/novobiocin	≤1/2	≤1/2	0 (0)^c^
Lincosamides	Pirlimycin	>4	>4	84 (75.0)^a^
Macrolides	Erythromycin	>4	>4	85 (75.9)^a^
Sulfonamides	Tetracycline	>8	>8	91 (81.3)^a^
Tetracyclines	Sulfadimethoxine	>256	>256	–^*b*^

^*a*^ Different superscript letters within a column indicate significant differences (*P* < 0.05, Bonferroni-corrected).

^*b*^ –, resistance could not be determined.

### Antimicrobial resistance genes in *S. uberis*

Antimicrobial resistance genes in the *S. uberis* isolates were detected using polymerase chain reaction (PCR) (Table 2). The most frequently identified gene was *tetO* (82.1%, *n* = 92), followed by *ermB* (78.6%, *n* = 88). *ermC* and *linB* were detected in two (1.8%) and one (0.9%) isolates, respectively. *ermA*, *tetS*, and *tetM* were not detected in any of the isolates.

**TABLE 2:** Antimicrobial resistance phenotypes and the corresponding resistance genes of *Streptococcus uberis* isolates from bovine mastitis milk^*b*^

Antimicrobial resistance	*N* (%)	Resistance genes						
		Lincosamides	Macrolides			Tetracyclines		
		*linB*	*ermA*	*ermB*	*ermC*	*tetM*	*tetO*	*tetS*
ERY/PIRL/TET^*a*^	80 (71.4)	1	0	80	2	0	80	0
Pan-susceptible	20 (17.9)	0	0	1	0	0	0	0
TET	6 (5.4)	0	0	3	0	0	6	0
ERY/OXA/PIRL/TET^*a*^	3 (2.7)	0	0	3	0	0	3	0
ERY/PIRL	1 (0.9)	0	0	1	0	0	1	0
ERY/TET	1 (0.9)	0	0	1	0	0	1	0
OXA/TET	1 (0.9)	0	0	0	0	0	1	0
No. of gene-positive isolates (%)		1 (0.9)	0 (0)	89 (79.5)	2 (1.8)	0 (0)	82 (82.1)	0 (0)

^*a*^ Multidrug resistance.

^*b*^ ERY, erythromycin; *N*, number of isolates exhibiting antimicrobial resistance phenotype patterns; OXA, oxacillin + 2% NaCl; PIRL, pirlimycin; TET, tetracycline.

The distribution pattern of antimicrobial resistance genes among the isolates showed that *ermB*/*tetO* was the most common combination, detected in 85 isolates (75.9%), followed by isolates in which no targeted resistance genes were detected (19 isolates, 17.0%). *tetO* was identified in four isolates (3.6%), *ermB*/*ermC*/*tetO* in two (1.8%), and *ermB* and *ermB*/*linB*/*tetO* in one isolate each (0.9%).

The associations between antimicrobial resistance genes and resistance phenotypes were evaluated using Fisher’s exact test. A significant association was observed between the presence of *tetO* and tetracycline resistance, as well as between *ermB* and erythromycin and pirlimycin resistance (*P* < 0.0001 for all comparisons) (Table 3).

**TABLE 3:** Association between antimicrobial resistance genes and resistance phenotypes in 112 *Streptococcus uberis* isolates from bovine mastitis milk

Resistance gene	Antimicrobial phenotype	Gene-positive resistant isolates, *n* (%)	Gene-negative resistant isolates, *n* (%)	*P* value
*tetO*	Tetracycline	91/92 (98.9%)	0/20 (0%)	<0.0001
*ermB*	Erythromycin	85/89 (95.5%)	0/23 (0%)	<0.0001
*ermB*	Pirlimycin	84/89 (94.4%)	0/23 (0%)	<0.0001

### Virulence genes in *S. uberis*

The virulence-associated genes in the *S. uberis* isolates were assessed using PCR to determine their distribution patterns (Table 4). *mtu*, *oppF*, and *sua* were detected in all isolates. *gapC* was identified in 111 isolates (99.1%). *pauA* and *skc* were detected in 100 isolates each (89.3%). *hasC* was found in 78 isolates (69.6%), while *cfu* and *lbp* were detected in 45 (40.2%) and 24 isolates (21.4%), respectively. *hasA* and *hasB* were identified in 11 isolates each (9.8%).

**TABLE 4:** Virulence gene distribution and biofilm formation in *Streptococcus uberis* isolates from bovine mastitis milk^*a*^

Virulence gene		No. of isolates (%)
CAMP factor	*cfu*	45 (40.2)^d^
Surface dehydrogenase protein	*gapC*	111 (99.1)^a^
Hyaluronic acid capsule and resistance to phagocytosis	*hasA*	11 (9.8)^f^
	*hasB*	11 (9.8)^f^
	*hasC*	78 (69.6)^c^
Lactoferrin binding protein	*lbp*	24 (21.4)^e^
Extracellular manganese-binding protein	*mtu*	112 (100)^a^
Oligopeptide transport ATP-binding protein	*oppF*	112 (100)^a^
Plasminogen activator	*pauA*	100 (89.3)^b^
Streptokinase activator	*skc*	100 (89.3)^b^
Adhesion molecule	*sua*	112 (100)^a^

Biofilm formation	No. of isolates (%)
Weak	101 (90.2)
Moderate	9 (8.0)
Strong	2 (1.8)

^*a*^ Different superscript letters within a column indicate significant differences (*P* < 0.05, Bonferroni-corrected).

### Biofilm-formation ability of *S. uberis*

The biofilm-formation ability of the *S. uberis* isolates was assessed using the crystal violet microtiter plate assay (Table 4). All isolates formed biofilms. The absorbance values for biofilm formation are shown in Fig. S1. Most isolates were classified as weak biofilm formers (90.2%). Two isolates (1.8%) exhibited strong biofilm-formation ability, whereas nine isolates (8.0%) exhibited moderate biofilm-formation ability.

## DISCUSSION

*Streptococcus uberis* has emerged as an important environmental pathogen associated with bovine mastitis, and thus, understanding its molecular and phenotypic traits has become increasingly relevant (2, 4). In this study, we investigated the molecular and phenotypic characteristics of 112 *S. uberis* isolates recovered from bovine mastitis quarter-milk samples collected across South Korea between 2019 and 2024. We integrated analyses of genetic relatedness, antimicrobial resistance, virulence-associated genes, and biofilm-formation ability.

The *S. uberis* isolates retrieved from dairy farms across South Korea demonstrated considerable genetic diversity, with multiple novel alleles; all STs were newly assigned. This high level of diversity is consistent with previous reports describing substantial heterogeneity within this species and does not support the expansion of a single dominant clone in the present population (11–15). The SLV-based goeBURST analysis further supported this observation as most STs formed small clusters or appeared as singletons. In previous global analyses, many *S. uberis* isolates have been assigned to broad lineage groups, such as the ST-5, ST-86, and ST-143 complexes, with the ST-5 and ST-143 complexes reported as major contributors to bovine mastitis in several countries (3, 21). Although 55.6% of the isolates in this study were assigned to the broad ST-143 complex, the absence of SLV connections to previously reported global profiles suggests that the South Korean strains represent divergent or previously unsampled branches within this lineage. In addition, the ST-05 complex, a major bovine mastitis-associated lineage in Europe, was not detected in our isolates (9). Although MLST alone cannot determine the ecological drivers underlying this diversity, the observed population structure may reflect the influence of multiple environmental or herd-level factors. Future studies incorporating whole-genome sequencing and more systematic sampling will be necessary to clarify the evolutionary relationships and ecological dynamics underlying these diverse lineages.

Antimicrobial administration remains the primary treatment approach for bovine mastitis, one of the most common indications for antimicrobial use in dairy cows (3, 5). However, inappropriate or excessive antimicrobial use may contribute to the development of antimicrobial resistance and facilitate the dissemination of resistant bacteria into the food chain, raising public health concerns (3). In the present study, the highest resistance rate was observed for tetracycline, followed by erythromycin and pirlimycin. All isolates remained susceptible to β-lactam antibiotics, including penicillin, ampicillin, ceftiofur, and cephalothin. These findings are consistent with those of previous studies, describing frequent tetracycline resistance and a low prevalence of β-lactam resistance among *S. uberis* isolates (20, 22–25). In South Korea, β-lactams are the most commonly used antimicrobial agents in cattle, followed by macrolides and tetracyclines, according to recent national veterinary antimicrobial sales data (26). These usage patterns may partially explain the resistance profiles observed in this study, particularly the relatively high resistance of *S. uberis* isolates to tetracycline, erythromycin, and pirlimycin. In contrast, despite their widespread use, the absence of resistance to β-lactams may be explained by the distinct resistance mechanisms in streptococci. Unlike resistance to tetracyclines and macrolides, which is often mediated by horizontally transferable genes, reduced susceptibility to β-lactams in streptococci is primarily associated with alterations in penicillin-binding proteins and involves a complex, multifactorial process driven by the accumulation of genetic changes (27, 28). These characteristics may limit the dissemination of β-lactam resistance in *S. uberis*. In addition, 74.1% of mastitis-associated *S. uberis* isolates were classified as multidrug-resistant in this study. This finding is broadly consistent with previous reports from South Korea, although considerable variation between countries has been described (20). Multidrug resistance has been reported in all isolates in Egypt, 58.6% of isolates in China, and 15.0% of isolates in Germany, highlighting substantial geographic heterogeneity in resistance burden (14, 19, 22). Such variations may be influenced by differences in antimicrobial use policies, herd management practices, livestock production systems, and the regional population dynamics of *S. uberis* (23). Therefore, continued surveillance and prudent antimicrobial use are essential to limit the dissemination of antimicrobial resistance in dairy herds.

The strong associations observed between resistance genes (*tetO* and *ermB*) and their corresponding phenotypes demonstrated a high level of genotype–phenotype concordance among the *S. uberis* isolates examined in this study. All tetracycline-resistant isolates in this study carried *tetO. tetO* encodes a ribosomal protection protein that enables translation to proceed in the presence of tetracyclines (29). Thus, its consistent detection suggests that the translation mechanism contributes to tetracycline resistance in *S. uberis* isolates from bovine mastitis in South Korea. In addition, 84 isolates (75.0%) in this study exhibited concurrent resistance to erythromycin and pirlimycin, and all of these isolates carried *ermB*. The coexistence of erythromycin and pirlimycin resistance in isolates carrying *ermB* suggests an MLS-type resistance mechanism, in which methylation of 23S rRNA reduces antibiotic binding to the ribosomal target site, thereby conferring reduced susceptibility to macrolides and lincosamides (21). This observation is consistent with that of a previous study, identifying *ermB* as a major determinant of macrolide and lincosamide resistance in mastitis-associated *S. uberis* (30). Furthermore, previous studies have demonstrated that tetracycline- and macrolide-resistance genes, including *tetO* and *ermB*, are frequently associated with integrative and conjugative elements, indicating their potential for horizontal transfer as linked genetic units (29, 31, 32). The consistent co-occurrence of *tetO* and *ermB* observed in this study supports the possibility that mobile genetic element-mediated transfer contributes to the persistence and spread of combined tetracycline and MLS resistance in mastitis-associated *S. uberis*. However, the genetic context of these resistance determinants was not directly examined in the present study. Their co-occurrence highlights the need for further investigation into the genetic linkage and transmission dynamics of resistance genes in this species, potentially through whole genome sequencing-based approaches.

In the present study, most isolates (89.3–100%) harbored *gapC*, *mtuA*, *oppF*, *pauA*, *skc*, and *sua*, which are considered core virulence-associated determinants of *S. uberis*. These genes encode proteins involved in host adhesion (*gapC* and *sua*), nutrient acquisition and transport (*mtuA* and *oppF*), immune evasion (*skc*), and host protein interaction (*pauA*), thereby facilitating colonization and persistence within the bovine mammary gland (19, 33). The high prevalence of these genes indicates that most isolates share a conserved virulence gene repertoire associated with intramammary infection. This finding is consistent with that of previous studies from China, Thailand, and the Czech Republic, while slightly lower prevalence rates (61.5–83.3%) have been reported in isolates from Argentina (14, 17, 18, 34). In contrast, capsule- and surface-associated virulence genes showed a heterogeneous distribution among isolates in this study. The hyaluronic acid capsule biosynthesis genes *hasA* and *hasB* were detected at low frequencies (9.8% each) (33). The prevalence of *hasA* and *hasB* observed in the present study was lower than that recorded in previous studies from China, Thailand, Argentina, and the Czech Republic, in which the detection rates ranged from 40.6% to 94% and from 66.6% to 96.4%, respectively (14, 17, 18, 34). *hasC* was identified in 69.6% of our isolates, which was comparable to the prevalence range reported by these studies (62.0–100%) (14, 17, 18, 34). In addition, *cfu* and *lbp* were detected at frequencies of 40.2% and 21.4%, respectively, in our isolates. Previous studies conducted in different countries have also reported variable prevalence rates for these genes (14, 17–19, 34). This distribution suggests that virulence-associated functions related to intramammary infection are widely maintained across genetically diverse *S. uberis* isolates. In contrast, the variability observed in capsule- and surface-associated genes indicates that these traits are not uniformly distributed within the population.

Biofilm formation is recognized as an important virulence-associated trait in *S. uberis*, as the biofilm matrix facilitates bacterial persistence within the mammary gland by enhancing protection against host immune responses (2, 16). In our study, all isolates were capable of forming biofilms, although most (90.2%) demonstrated only weak biofilm-formation ability. Comparable distributions have been reported by studies from China and South Africa, in which all mastitis-associated isolates exhibited biofilm-formation ability, with a dominant proportion of weak biofilm formers (14, 35). Findings of a study from Argentina further indicate that weak yet detectable biofilm formation may represent a common phenotypic characteristic of field-derived *S. uberis* populations (36). Taken together, these observations indicate that biofilm formation is a widely conserved phenotypic trait among *S. uberis* isolates, despite generally low levels of biofilm production. The predominance of weak biofilm formers in this study may suggest that high-level biofilm production is not required for persistence within the mammary environment. This widespread biofilm-forming ability suggests that biofilm formation may contribute to bacterial persistence within the udder environment and potentially to the recurrence or chronicity of intramammary infections. However, because biofilm formation was assessed under *in vitro* conditions, further studies are needed to determine how *in vitro* biofilm-forming ability relates to persistence within the bovine mammary gland.

In conclusion, *S. uberis* isolates recovered from bovine mastitis milk samples in South Korea were characterized by substantial genetic diversity, with no evidence of a dominant clonal lineage. High levels of resistance to tetracycline, erythromycin, and pirlimycin were observed, and multidrug resistance was commonly detected among the isolates. The strong association between resistance phenotypes and the presence of *tetO* and *ermB* highlights the importance of ribosomal protection and MLS-type resistance in these isolates. Virulence-associated genes involved in adhesion, nutrient acquisition, immune evasion, and host interaction were widely detected, whereas capsule- and surface-associated virulence genes showed heterogeneous distribution. Moreover, the widespread ability of the isolates to form biofilm may contribute to bacterial persistence within the mammary gland. Therefore, these findings contribute to a better understanding of the epidemiology and pathogenic potential of *S. uberis* associated with bovine mastitis in South Korea, thus highlighting the importance of continued surveillance and prudent antimicrobial use.

## MATERIALS AND METHODS

### Isolation and identification of *S. uberis*

As part of the National Bovine Mastitis Control Program in South Korea, a total of 112 *S. uberis* isolates were collected from regional laboratories and centers across the country. These isolates were retrieved from quarter milk samples obtained from lactating cows that were suspected of having either clinical or subclinical mastitis between 2019 and 2024. Bacterial isolation was performed following the procedures recommended by the National Mastitis Council guidelines (37). The isolates were forwarded to the Animal and Plant Quarantine Agency for further microbiological characterization. Species were confirmed using matrix-assisted laser desorption/ionization time-of-flight mass spectrometry (MALDI-TOF MS; bioMérieux, Marcy-l’Étoile, France) according to the manufacturer’s instructions. All isolates were preserved at −80°C until use.

### MLST and phylogenetics

MLST of *S. uberis* isolates was carried out according to the protocol described on the *S. uberis* MLST website (https://pubmlst.org/organisms/streptococcus-uberis) using seven housekeeping genes (*arcC*, *ddl*, *gki*, *recP*, *tdk*, *tpi*, and *yqiL*) (10). Allele numbers and STs were assigned based on the PubMLST *S. uberis* database.

A phylogenetic tree was constructed from the concatenated sequences of the seven loci using the MEGA11 software. The tree was constructed using the neighbor-joining method with the Tamura-Nei substitution model, and the robustness of the branches was assessed with 1,000 bootstrap replications.

For comparison with global *S. uberis* populations, additional MLST profiles (*n* = 467) were retrieved from the PubMLST database, restricted to isolates with the source listed as cow, disease as mastitis, and sample type as milk (Table S1). These reference profiles were analyzed together with the isolates obtained in this study. The relationships among STs were further visualized using the goeBURST algorithm implemented in PHYLOViZ 2.0. A minimum spanning tree was generated at the SLV level, in which nodes represent unique STs and edges connect STs differing at a single locus. The node size was proportional to the number of isolates, and the nodes were color-coded by country of origin to illustrate the geographic distribution of *S. uberis* lineages.

### Antimicrobial susceptibility testing

The antimicrobial susceptibility of *S. uberis* isolates was determined using the broth microdilution method in accordance with the Clinical and Laboratory Standards Institute (CLSI) guideline M100 (38). MICs were obtained with a Sensititre CMV1AMAF panel (Trek Diagnostics, Cleveland, OH, USA) following the manufacturer’s recommendations. The panel comprised 10 antimicrobial agents at various concentration ranges (μg/mL): ampicillin (0.12–8), cephalothin (2–16), ceftiofur (1–4), penicillin (0.12–8), penicillin–novobiocin combination (1/2–8/16), pirlimycin (0.5–4), sulfadimethoxine (32–256), erythromycin (0.25–4), oxacillin + 2% NaCl (2–4), and tetracycline (1–8). MIC values were interpreted based on CLSI guidelines and previous studies (38–41). *Streptococcus pneumoniae* ATCC 49619 was used as the quality control strain to verify the accuracy of susceptibility testing. Isolates exhibiting resistance to three or more antimicrobial classes were classified as multidrug-resistant.

### Detection of virulence and antimicrobial-resistance genes

Genomic DNA from *S. uberis* isolates was extracted using the boiling method as described previously (42). The isolates were screened using PCR for the presence of virulence-associated and antimicrobial-resistance genes. The virulence genes analyzed included those associated with CAMP factor (*cfu*), surface dehydrogenase protein (*gapC*), hyaluronic acid capsule and resistance to phagocytosis (*hasA*, *hasB*, and *hasC*), lactoferrin-binding protein (*lbp*), extracellular manganese‐binding protein (*mtu*), oligopeptide transport ATP‐binding protein (*oppF*), plasminogen activator (*pauA*), streptokinase activator (*skc*), and adhesion molecule (*sua*). Whereas, antimicrobial-resistance genes targeted classes corresponding to lincosamides (*linB*), macrolides (*ermA*, *ermB*, and *ermC*), and tetracyclines (*tetM*, *tetO*, and *tetS*). Primer sequences and PCR amplification conditions used for gene detection are listed in Table S2. The PCRs were performed under standard conditions, as described in previous reports (6, 14, 18, 33, 43–48). The PCR assays were performed using previously characterized gene-positive strains as positive controls and distilled water as a negative control. Representative amplicons were sequenced to confirm the identity of the target genes.

### Biofilm assay

The biofilm‐formation ability of *S. uberis* isolates was assessed using a microtiter plate-based crystal violet assay, following previously established procedures with minor modifications (14). A single colony of each isolate was inoculated in 5 mL of tryptic soy broth and incubated at 37°C for 24 h. The overnight culture was subsequently diluted 1:50 in fresh brain heart infusion broth, and 200 µL of the diluted suspension was dispensed into each well of sterile 96-well microtiter plates. The plates were incubated at 37°C for 48 h to allow biofilm development. Following incubation, the contents of the wells were washed three times with phosphate-buffered saline (PBS) to remove non-adherent cells. Biofilms attached to the well surface were stained with 200 µL of 0.2% crystal violet for 15 min. Excess stain was removed by washing the well contents three more times with PBS, and the plates were air-dried. The bound crystal violet was then solubilized with 200 µL of 95% ethanol for 1 h. A 100 µL aliquot of the extracted stain from each well was transferred to a new plate, and the absorbance was measured at 595 nm. Uninoculated BHI medium was used as a negative control. *Streptococcus pneumoniae* ATCC 49619 was included as a reference strain to validate the assay. Biofilm-formation ability of the isolates was evaluated relative to that of these controls. Biofilm-formation ability was classified according to the optical density (OD) values relative to the negative control as follows: non-adherent (OD ≤ OD of the negative control), weak (OD of the negative control <OD ≤ 2 × OD of the negative control), moderate (2 × OD of the negative control <OD ≤ 4 × OD of the negative control), or strong (OD >4 × OD of the negative control) (49).

### Statistical analysis

Categorical variables were analyzed using Pearson’s chi-squared test or Fisher’s exact test, as appropriate. Differences in resistance rates to antibiotics and in detection rates of virulence-associated genes were evaluated using these tests, followed by Bonferroni correction for multiple comparisons. The association between antimicrobial-resistance phenotypes and resistance genes was also evaluated using Fisher’s exact test. Statistical significance was set at *P* < 0.05. All analyses were performed using SPSS Statistics version 25.0 (IBM Corp., Armonk, NY, USA).

## Data Availability

MLST data generated in this study have been deposited in the PubMLST database. All other data supporting the findings of this study are available within the article and its supplemental material.

## SUPPLEMENTAL MATERIAL

Fig. S1 spectrum.00772-26-s0001.docx Biofilm formation by *Streptococcus uberis* isolates from bovine mastitis milk.

Table S1 spectrum.00772-26-s0002.xlsx MLST profiles of *Streptococcus uberis* isolates retrieved from the PubMLST database used for global comparison.

Table S2 spectrum.00772-26-s0003.docx Primers used in this study.

ASM does not own the copyrights to Supplemental Material that may be linked to, or accessed through, an article. The authors have granted ASM a non-exclusive, world-wide license to publish the Supplemental Material files. Please contact the corresponding author directly for reuse.
