# Supplementary material for: Molecular and phenotypic characterization of Streptococcus uberis isolates from bovine mastitis milk in South Korea
Source: Microbiol Spectr. 2026 Jun 22;14(8):e00772-26. doi: 10.1128/spectrum.00772-26 (PMC13436408; doi:10.1128/spectrum.00772-26)
Supplement: Supplementary file 1 [file spectrum.00772-26-s0001.docx]

**Figure S1. Biofilm formation by *Streptococcus uberis* isolates from bovine mastitis milk**

Biofilm formation was quantified by crystal violet staining assay and expressed as optical density values. Data are presented as mean ± standard deviation.
