# Supplementary material for: Molecular and phenotypic characterization of Streptococcus uberis isolates from bovine mastitis milk in South Korea
Source: Microbiol Spectr. 2026 Jun 22;14(8):e00772-26. doi: 10.1128/spectrum.00772-26 (PMC13436408; doi:10.1128/spectrum.00772-26)
Supplement: Supplementary file 2 [file spectrum.00772-26-s0003.docx]

Table S2. Primers used in this study

| **Gene** | **Primer sequence (5’-3’)** | **Annealing**  **temperature** | **Amplicon**  **size (bp)** | **Reference** |
| --- | --- | --- | --- | --- |
| **Antimicrobial**  **resistance gene** |  |  |  |  |
| *ermA* | F: GTTCAAGAACAATCAATACAGAG | 48 | 421 | (44) |
|  | R: GGATCAGGAAAAGGACATTTTAC |  |  |  |
| *ermB* | F: AAAGCCATGCGTCTGACATC | 55 | 194 | (42) |
|  | R: TGTGGTATGGCGGGTAAGTT |  |  |  |
| *ermC* | F: ATCTTTGAAATCGGCTCAGG | 47 | 295 | (43) |
|  | R: CAAACCCGTATTCCACGATT |  |  |  |
| *linB* | F: CCTGATACGAAGGCTATGCTT | 55 | 403 | (42) |
|  | R: GGTGACTTTGCAAATCCATAACT |  |  |  |
| *tetS* | F: AGGACAAACTTTCTGACGACA | 55 | 306 | (42) |
|  | R: CTGAATTGAGTTGTGTGGGTGA |  |  |  |
| *tetM* | F: GCGTACAAGCACAAACTCGT | 54 | 201 | (14) |
|  | R: CGGTAAAGTTCGTCACACACAT |  |  |  |
| *tetO* | F: ATATGGGGATGCTGCCCAAC | 54 | 349 | (14) |
|  | R: TGGAGCGTCAAAGGGGAATC |  |  |  |
| **Virulence gene** |  |  |  |  |
| *hasA* | F: GAAAGGTCTGATGCTGATG | 58 | 445 | (40) |
|  | R: TCATCCCCTATGCTTACAG |  |  |  |
| *hasB* | F: TCTAGACGCCGATCAAGC | 58 | 319 | (40) |
|  | R: TGAATTCCTATGCGTCGATC |  |  |  |
| *hasC* | F: TGCTTGGTGACGATTTGATG | 58 | 532 | (40) |
|  | R: GTCCAATGATAGCAAGGTCAC |  |  |  |
| *gapC* | F: GCTCCTGGTGGAGATGATGT | 55 | 200 | (18) |
|  | R: GTCACCAGTGTAAGCGTGGA |  |  |  |
| *lbp* | F: CGACCCTTCAGATTGGATTC | 55 | 698 | (18) |
|  | R: TAGCAGCATCACGTTCTTCG |  |  |  |
| *pauA* | F: TGCTACTCAACCATCAAAGGTTGC | 57 | 439 | (39) |
|  | R: TAGCAGTCTCAGTAGGATGAGTGA |  |  |  |
| *oppF* | F: GGCCTAACCAAAACGAAACA | 54 | 419 | (41) |
|  | R: GGCTCTGGAATTGCTGAAAG |  |  |  |
| *sua* | F: TCAACTTGACGAATCGCTTG | 53 | 480 | (6) |
|  | R: TCAGCCATTGTTTCTGCTTG |  |  |  |
| *mtuA* | F: GAACCGCTACCTGAGGATGT | 54 | 500 | (29) |
|  | R: ATTTGATCCGGTGTTCCTTC |  |  |  |
| *cfu* | F: TATCCCGATTTGCAGCCTAC | 50 | 205 | (18) |
|  | R: CCTGGTCAACTTGTGCAACTG |  |  |  |
| *skc* | F: TCCGGATTTTGGGTCCTTAGCCA | 52 | 475 | (39) |
|  | R: AGTCGACTTTGCGCCTGATGCAC |  |  |  |
